# Solving Program Sketches with Large Integer Values

**DOI:** 10.1007/978-3-030-44914-8_21

**Published:** 2020-04-18

**Authors:** Rong Pan, Qinheping Hu, Rishabh Singh, Loris D’Antoni

**Affiliations:** ETH Zurich, Zurich, Switzerland; 9 The University of Texas at Austin, Austin, USA; 10 University of Wisconsin-Madison, Madison, USA; 11 Google, Mountain View, USA

## Abstract

Program sketching is a program synthesis paradigm in which the programmer provides a partial program with holes and assertions. The goal of the synthesizer is to automatically find integer values for the holes so that the resulting program satisfies the assertions. The most popular sketching tool, Sketch, can efficiently solve complex program sketches, but uses an integer encoding that often performs poorly if the sketched program manipulates large integer values. In this paper, we propose a new solving technique that allows Sketch to handle large integer values while retaining its integer encoding. Our technique uses a result from number theory, the Chinese Remainder Theorem, to rewrite program sketches to only track the remainders of certain variable values with respect to several prime numbers. We prove that our transformation is sound and the encoding of the resulting programs are exponentially more succinct than existing Sketch encodings. We evaluate our technique on a variety of benchmarks manipulating large integer values. Our technique provides speedups against both existing Sketch solvers and can solve benchmarks that existing Sketch solvers cannot handle.

### 1 Introduction

Program synthesis, the art of automatically generating programs that meet a user's intent, promises to increase the productivity of programmers by automating tedious, error-prone, and time-consuming tasks. Syntax-guided Synthesis (SyGuS) [2], where the search space of possible programs is defined using a grammar or a domain-specific language, has emerged as a common program synthesis paradigm for many synthesis domains. One of the earliest and successful syntax-guided program synthesis frameworks is program sketching [19], where $(i)$ the search space of the synthesis problem is described using a partial program in which certain integer constants are left unspecified (represented as holes), and $(ii)$ the specification is provided as a set of assertions describing the intended behavior of the program. The goal of the synthesizer is to automatically replace the holes in the program with integer values so that the resulting complete program satisfies all the assertions. Thanks to its simplicity, program sketching has found wide adoption in applications such as data-structure design [20], personalized education [18], program repair [7], and many others.

The most popular sketching tool, SKETCH [21], can efficiently solve complex program sketches with hundreds of lines of code. However, SKETCH often performs poorly if the sketched program manipulates large integer values. SKETCH's synthesis is based on an algorithm called *counterexample-guided inductive synthesis* (CEGIS) [21]. The CEGIS algorithm iteratively considers a finite set $I$ of inputs for the program and performs SAT queries to identify values for the holes so that the resulting program satisfies all the assertions for the inputs in $I$. Further SAT queries are then used to verify whether the generated solution is correct on all the possible inputs of the program. SKETCH represents integers using a unary encoding (a variable for each integer value) so that arithmetic computations such as addition, multiplication etc. can be represented efficiently in the SAT formulas as lookup operations. This unary encoding, however, results in huge formulas for solving sketches with larger integer values as we also observe in our evaluation. Recently, an SMT-like technique that extends the SAT solver with native integer variables and integer constraints was proposed to alleviate this issue in SKETCH. It guesses values for the integer variables and propagates them through the integer constraints, and learns from conflict clauses. However, this technique does not scale well when the sketches contain complex arithmetic operations—e.g., non-linear integer arithmetic.

In this paper, we propose a program transformation technique that allows SKETCH to solve program sketches involving large integer values while retaining the unary encoding used by the traditional SKETCH solver. Our technique rewrites a SKETCH program into an equivalent one that performs computations over smaller values. The technique is based on the well-known Chinese Remainder Theorem, which states that, given distinct prime numbers $p_1, \ldots, p_n$ such that $N = p_1 \cdot \ldots \cdot p_n$, for every two distinct numbers $0 \leq k_1, k_2 < N$, there exists a $p_i$ such that $k_1 \bmod p_i \neq k_2 \bmod p_i$. Intuitively, this theorem states that tracking the modular values of a number smaller than $N$ for each $p_i$ is enough to uniquely recover the actual value of the number itself. We use this idea to replace a variable $x$ in the program with $n$ variables $x_{p_1}, \ldots, x_{p_n}$, so that for every $i$, $x_{p_i} = x \bmod p_i$. Using closure properties of modular arithmetic we show that, as long as the program uses the operators $+, -, *, ==$, tracking the modular values of variables and performing the corresponding operations on such values is enough to ensure correctness. For example, to reflect the variable assignment $x = y + z$, we perform the assignment $x_{p_i} = (y_{p_i} + z_{p_i}) \bmod p_i$, for every $p_i$. Similarly, the Boolean operation $x == y$ will only hold if $x_{p_i} = y_{p_i}$, for every $p_i$. To identify what variables and values in the program can be rewritten, we develop a data-flow analysis that computes what variables *may* flow into operations that are not sound in modular arithmetic—e.g., $<, >, \leq$, and $/$.

We provide a comprehensive theoretical analysis of the complexity of the proposed transformation. First, we derive how many prime numbers are needed to track values in a certain integer range. Second, we analyze the number of bits required to encode values in the original and rewritten program and show that, for the unary encoding used by SKETCH, our technique offers an exponential saving in the number of required bits.

We evaluate our technique on 181 benchmarks from various applications of program sketching. Our results show that our technique results in significant speedups over existing SKETCH solvers and is able to solve 48 benchmarks on which SKETCH times out.

*Contributions.* In summary, our contributions are:

- A language IMP-MOD together with a *modular semantics* that represents integer values using their remainders for a given set of primes and a proof that this semantics is equivalent to the standard *integer semantics* (§ 4).
- A data-flow analysis for detecting variables that can be soundly executed in the modular semantics and an algorithm for translating IMP programs into IMP-MOD ones (§ 5).
- A synthesis algorithm for IMP-MOD programs and incremental synthesis algorithm that lazily increases the number of primes used in the modular semantics (§ 6).
- A complexity analysis that shows that synthesis for IMP-MOD programs requires exponentially smaller SAT queries than synthesis in IMP (§ 7).
- An evaluation of our technique on 181 benchmarks that manipulate large integer values. Our solver outperforms the default SKETCH unary solver, it can solve 48 new benchmarks that no SKETCH solver can solve, and is 15.9X faster than the SKETCH SMT-like integer solver on the hard benchmarks that take more than 10 seconds to solve (§ 8).

An extended version containing all proofs and further details has been uploaded to arXiv as supplementary material.

### 2 Motivating Example

In this section, we use a simple example to illustrate our technique and its effectiveness. Consider the SKETCH program `polyArray` presented in Figure 1b. The goal of this synthesis problem is to synthesize a two-variable quadratic polynomial (lines 7–8) whose evaluation `p` on given inputs `x` and `y` is equal to a given expected-output array `z` (line 9). Solving the problem amounts to finding non-negative integer values for the holes (`??`) and sign values, i.e., -1 or 1, for the holes (`??`$^s$) such that the assertion becomes true.[1] In this case, a possible solution is the polynomial:

```
p[i] = -17*y[i]^2-8*x[i]*y[i]-17*x[i]^2-3*x[i];
```

When attempting to solve this problem, the SKETCH synthesizer times out at 300 seconds. To solve this problem, SKETCH creates SAT queries where the variables are the holes. Due to the large numbers involved in the computation of this program, the unary encoding of SKETCH ends up with SAT formulas with approximately 45 *million* clauses.

[1] In SKETCH, holes can only assume positive values. This is why we need the sign holes, which are implemented using regular holes as follows: `if(??) then 1 else -1`.

```
// n=4, x=[24,-1,0,-19], y=[-7,11,-3,13]
// z=[-9353,-1983,-153,-6977]
polyArray(int n, int[n] x, int[n] y, int[n] z){
  int[n] p;
  int i=0;
  while (i<n){
    p[i]=??_1^s*??_1*y[i]^2+??_2^s*??_2*x[i]^2+??_3^s*??_3*x[i]*y[i]
         +??_4^s*??_4*y[i]+??_5^s*??_5*x[i]+??_6^s*??_6;
   assert p[i] == z[i];
   i++; }
}
```

(a) Original sketch program.

```
// n=4, x=[24,-1,0,-19], y=[-7,11,-3,13]
// z=[-9353,-1983,-153,-6977]
pAPrime(int n, int[n] x, int[n] y, int[n] z){
  int[n] x2,x3,x5,x7,x11,x13,x17;
  while (i<n){   // Initialize modular variables
    x2[i]=x[i]%2;
    x3[i]=x[i]%3;
    ... i++; }
  int i=0;
  int[n] p2,p3,p5,p7,p11,p13,p17;
  while (i<n){
    p2[i]=(??_1^s*(??_1%2)*(y2[i]^2%2)%2
          +??_2^s*(??_2%2)*(x2[i]^2%2)%2
          +??_3^s*(??_3%2)*(x2[i]%2)*(y2[i]%2)%2
          +??_4^s*(??_4%2)*(y2[i]%2)%2
          +??_5^s*(??_5%2)*(x2[i]%2)%2
          +??_6^s*(??_6%2)%2)%2;
    ...
    assert p2[i] = z2[i];
    assert p3[i] = z3[i];
    ...
    i++; }
}
```

(b) Rewritten sketch program.

Fig. 1: SKETCH program (a) and rewritten version with values tracked for different moduli (b).

*Sketch Program with Modular Arithmetic* The technique we propose in this paper has the goal of reducing the complexity of the synthesis problem by transforming the program into an equivalent one that manipulates smaller integer values and that yields easier SAT queries. Given the SKETCH program in Figure 1b, our technique produces the modified SKETCH program `pAPrime` in Figure 1a. The new SKETCH program has the same control flow graph as the original one, but instead of computing the actual values of the expressions `x[·]` and `y[·]`, it tracks their remainders for the set of prime numbers $\{2, 3, 5, 7, 11, 13, 17\}$ using new variables—e.g., `x2[i]` tracks the remainder of `x[i]` modulo 2.

The program `pAPrime` initializes the modular variables with the corresponding modular values (lines 5–8). When rewriting a computation over modular variables, the same computation is performed modularly (lines 12–17). For example, the term $\texttt{??}_1^s * \texttt{??}_1 \texttt{*y[i]}^2$ when tracked modulo 2 is rewritten as

$$\texttt{(??}_1^s\texttt{*(??}_1\texttt{\%2)*((y2[i]\%2)}^2\texttt{\%2))\%2}$$

In the rewritten program, the variables `i` and `n` are not tracked modularly, since such a transformation would incorrectly access array indices. Finally, the assertions for different moduli share the same holes as the solution to the Sketch has to be correct for all modular values. In the rest of the paper, we develop a data flow analysis that detects when variables can be tracked modularly.

Sketch can solve the rewritten program in less than 2 seconds and produce hole values that are correct solutions for the original program. This speedup is due to the small integer values manipulated by the modular computations. In fact, the intermediate SAT formulas generated by Sketch for the program `pAPrime` have approximately 120 *thousand* clauses instead of the 45 *million* clauses for `polyArray`. Due to the complex arithmetic in the formulas, even if Sketch uses the SMT-like native integer encoding, it still requires more than 300 seconds to solve this problem.

While this technique is quite powerful, it does have some limitations. In particular, the solution to the rewritten Sketch is guaranteed to be a correct solution only for inputs that cause intermediate values of the program to be in a range $[d_1, d_2]$ such that $d_2 - d_1 \leq 2 \times 3 \times 5 \times 7 \times 11 \times 13 \times 17 = 510,510$. We will prove this result in Section 4.

### 3 Preliminaries

In this section, we describe the `IMP` language that we will consider throughout the paper and briefly recall the counter-example guided inductive synthesis algorithm employed by the Sketch solver.

For simplicity, we consider a simple imperative language `IMP` with integer holes for defining the hypothesis space of programs. The syntax and semantics of `IMP` are shown in Appendix **??**. Without loss of generality, we assume the programs consists of a single program $f(v_1, \cdots, v_n, ??_1, \ldots ??_m)$ with $n$ integer variables and $m$ integer holes. The body of the program $f$ consists of a sequence of statements, where a statement $s$ can either be a variable assignment, a `while` loop statement, an `if` conditional statement, or an `assert` statement. The holes `??` denote integer constant values that are unknown and the goal of the synthesis process is to compute these values such that a set of desired program assertions are satisfied for every possible input values to $f$.[2]

[2] Our implementation also supports for-loops, recursion, arrays, and complex types.

*Example 1.* An example IMP sketch denoting a partial program is shown below.

```
triple(n,h,??){ h=??; assert h*n==n+n+n; }
```

The goal of the synthesizer is to compute the value of the hole ?? such that the assertion is true for all possible input values of n and h. For this example, $\texttt{??} = 3$ is a valid solution.

The SKETCH solver uses the counter-example guided inductive synthesis algorithm (CEGIS) to find hole values such that the desired assertions hold for all input values. Formally, the SKETCH synthesizer solves the following constraint:

$$\exists\texttt{??} \equiv (\texttt{??}_1, \cdots, \texttt{??}_m) {\in} \mathbb{Z}^m.\ \forall in {\in} \mathcal{I}.\ [\![ f(in, \texttt{??}) ]\!]^{\texttt{IMP}} \neq \bot$$

where $\mathbb{Z}$ denotes the domain of all integer values, ?? denotes the list of unknown hole values $(\texttt{??}_1, \cdots, \texttt{??}_m) \in \mathbb{Z}^m$, $\mathcal{I}$ denotes the domain of all input argument values to the function $f$, and $[\![ f(in, \texttt{??}) ]\!]^{\texttt{IMP}} \neq \bot$ denotes that the program satisfies all assertions. The synthesis problem is in general undecidable for a language with complex operations such as the IMP language because of the infinite size of possible hole and input values. To make the synthesis process more tractable, SKETCH imposes a bound on the sizes of both the input domain ($\mathcal{I}_b$) and the domain of hole values ($\mathbb{Z}_b$) to obtain the following constraint:

$$\exists\texttt{??} \equiv (\texttt{??}_1, \cdots, \texttt{??}_m) {\in} \mathbb{Z}_b^m.\ \forall in {\in} \mathcal{I}_b.\ [\![ f(in, \texttt{??}) ]\!]^{\texttt{IMP}} \neq \bot$$

The bounded domains make the synthesis problem decidable, but the second-order quantified formula results in a search space of hole values that is still huge for any reasonable bounds. To solve such bounded equations efficiently, SKETCH uses the CEGIS algorithm to incrementally add inputs from the domain until obtaining hole values ?? that satisfy the assertion predicates for all the input values in the bounded domain. The algorithm solves the second-order formula by iteratively solving a series of first-order queries. It first encodes the existential query (synthesis query) over a randomly selected input value $in_0$ to find the hole values $\boldsymbol{H}$ that satisfy the predicate for $in_0$ using a SAT solver in the backend.

$$\exists\texttt{??} \equiv (\texttt{??}_1, \cdots, \texttt{??}_m) \in \mathbb{Z}_b^m.\ [\![ f(in_0, \texttt{??}) ]\!]^{\texttt{IMP}} \neq \bot$$

It then encodes another existential query (verification) to now find a counter-example $in_1$ for which the predicate is not satisfied for the previously found hole values.

$$\exists in \in \mathcal{I}_b.\ \neg [\![ f(in, \boldsymbol{H}) ]\!]^{\texttt{IMP}} \neq \bot$$

If no counter-example input can be found, the hole values are returned as the desired solution. Otherwise, the algorithm computes a new hole value that satisfies the assertion for all the counter-example inputs found so far. This process continues iteratively until either a desired hole value is found (i.e. no counter-example input exists), no satisfiable hole value is found (i.e. the synthesis problem is infeasible), or the SAT solver times out.

*Integer Encoding* The SKETCH solver can efficiently solve the synthesis constraint in many domains, but it does not scale well for sketches manipulating large numbers. SKETCH uses a unary encoding to represent integers, where the encoded formula consists of a variable for each integer value. The unary encoding allows for simplifying the representation of complex non-linear arithmetic operations. For example, a multiplication operation can be represented as simply a lookup table using this encoding. In practice, the unary encoding results in magnitudes of faster solving times compared to the logarithmic encoding for many synthesis problems. However, this also results in huge SAT formulas in presence of large integers. Recently, a new SMT-like technique based on extending the SAT solver with native integer variables and constraints was proposed to alleviate this issue in SKETCH. Similar to the Boolean variables, this extended solver guesses for integer values and propagates them in the constraints while also learning from conflict clauses. Note that SKETCH uses these SAT extensions and encodings instead of an SMT solver as SMT doesn't scale well for the non-linear constraints typically found in the synthesis problems. Our new technique for handling computations over large numbers still maintains the efficient unary encoding of integers and computations over them.

## 4 Modular Arithmetic Semantics

In this section, we present the language `IMP-MOD` in which variables can be tracked using modular arithmetic. We start by recalling the Chinese Remainder Theorem, then define both a modular and integer semantics for the `IMP-MOD` language, and show that the two semantics are equivalent.

### 4.1 The Chinese Remainder Theorem

The Chinese Remainder Theorem is a powerful number theory result that shows the following: given a set of distinct primes $\mathbb{P} = \{p_1, \ldots, p_k\}$, any number $n$ in an interval of size $p_1 \cdot \ldots \cdot p_k$ can be uniquely identified from the remainders $[n \bmod p_1, \cdots, n \bmod p_k]$. In Section 4.2, we will use this idea to define the semantics of the `IMP-MOD` language. The main benefit of this idea is that the remainders could be much smaller than actual program values.

*Example 2.* For $\mathbb{P} = [3, 5, 7]$ and an integer 101, its remainders $[2, 1, 3]$ are much smaller than 101. However, any number of the form $101 + 105 \times n$ also has remainders $[2, 1, 3]$ with respect to the same prime set.

In general, one cannot uniquely determine an arbitrary integer value from its remainders for some set $\mathbb{P}$—i.e., the mapping from a number to its remainders is an abstraction in the sense of abstract interpretation [6]. However, if we are interested in a limited range of integer values $[L, U)$, one can choose a set of primes $\mathbb{P} = \{p_1, \ldots, p_k\}$ such that, for values $L \leq x < U$, the map $[r_1, \cdots, r_k] \mapsto x$, where $x \equiv r_i \bmod p_i$, is an injection.

$$\begin{aligned}
\text{Modular Expr } a^{\mathbb{P}} &:= c^{\mathbb{P}} \mid v^{\mathbb{P}} \mid a_1^{\mathbb{P}}\ op_a^{\mathbb{P}}\ a_2^{\mathbb{P}} \mid \textsc{toPrime}(a) \\
\text{Modular Op } op_a^{\mathbb{P}} &:= + \mid - \mid * \\
\\
\text{Arith Expr } a &:= \texttt{??} \mid c \mid v \mid a_1\ op_a\ a_2 \\
\text{Arith Op } op_a &:= + \mid - \mid * \mid / \\
\text{Bool Expr } b &:= \texttt{not}\ b \mid a_1\ op_c\ a_2 \mid b_1\ \texttt{and}\ b_2 \mid b_1\ \texttt{or}\ b_2 \mid a_1^{\mathbb{P}} {=}{=} a_2^{\mathbb{P}} \\
\text{Comp Op } op_c &:= < \mid > \mid \leq \mid \geq \\
\\
\text{Stmt } s &:= v = a \mid v^{\mathbb{P}} = a^{\mathbb{P}} \mid s_1; s_2 \\
&\mid \texttt{while}(b)\ \{s\} \mid \texttt{if}(b)\ s_1\ \texttt{else}\ s_2 \mid \texttt{assert}\ b \\
\text{Program } P &:= f\,(v_1, \cdots, v_n, v_1^{\mathbb{P}}, \cdots, v_m^{\mathbb{P}}, ??_1, \ldots, ??_l)\ \{s\}
\end{aligned}$$

Fig. 2: Syntax of the `IMP-MOD` language.

**Theorem 1 (Chinese Remainder Theorem [4]).** *Let $p_1$, ..., $p_k$ be positive integers that are pairwise co-prime—i.e., no two numbers share a divisor larger than 1. Denote $N = \prod_{i=1}^{k} p_i$, and let $d$, $r_1$, $r_2$, ..., $r_k$ be any integers. Then there is one and only one integer $d \leq x < d + N$ such that $x \equiv r_i \bmod p_i$ for every $1 \leq i \leq k$.*

We define the translation function $m_{\mathbb{P}}(x) := [x \bmod p_i, \cdots, x \bmod p_k]$ that maps an integer to its set of remainders with respect to $\mathbb{P}$. When $m_{\mathbb{P}}(x)$ is bijective on some set $R$, we denote with $m_{\mathbb{P}}^{-1,R} : [0, p_1) \times \cdots \times [0, p_k) \to R$ its inverse function.

*Example 3.* Let $x$ be a integer in the range $[0, 105)$ (note that $105 = 3 \times 5 \times 7$). If we know that the value of $x$ is congruent to $[2, 1, 3]$ modulo $\{3, 5, 7\}$, we can uniquely identify the value of $x$ to be 101 by observing that $101 \equiv 2 \bmod 3,\ 101 \equiv 1 \bmod 5,$ and $101 \equiv 3 \bmod 7$.

The following lemma shows that the function $m_{\mathbb{P}}$ is closed under addition, subtraction and multiplication of integers.

**Lemma 1.** *For every set of primes $\mathbb{P}$, integers $x$ and $y$, and $op \in \{+, -, *\}$, the following holds: $m_{\mathbb{P}}(x\ op\ y) = m_{\mathbb{P}}(x)\ op\ m_{\mathbb{P}}(y)$.*

#### 4.2 The `IMP-MOD` Language

In this section, we define the `IMP-MOD` language (syntax in Figure 2), a variant of the `IMP` language for which the semantics can be defined using modular arithmetic.[3] An `IMP-MOD` program is parametric on a set $\mathbb{P} = \{p_1, \ldots, p_k\}$ of distinct

[3] We consider the simple subset for a clear presentation of the semantics, but our framework works for the full `IMP` language (and for more complex language constructs) as we will see in the later sections.

$$[\![\textsc{toPrime}(a)]\!]^{\mathbb{P}}_{\sigma,\sigma^{\mathbb{P}}} := [\ [\![a]\!]^{\mathbb{P}}_{\sigma,\sigma^{\mathbb{P}}} \bmod p_1, \cdots\ ]$$

$$[\![v^{\mathbb{P}}]\!]^{\mathbb{P}}_{\sigma,\sigma^{\mathbb{P}}} := \sigma^{\mathbb{P}}(v) \qquad [\![c^{\mathbb{P}}]\!]^{\mathbb{P}}_{\sigma,\sigma^{\mathbb{P}}} := [\ c \bmod p_1, \cdots, c \bmod p_k\ ]$$

$$[\![a^{\mathbb{P}}_1\ op^{\mathbb{P}}_a\ a^{\mathbb{P}}_2]\!]^{\mathbb{P}}_{\sigma,\sigma^{\mathbb{P}}} := [\ (x^1_1\ op^{\mathbb{P}}_a\ x^2_1) \bmod p_1, \cdots\ ] \text{ where } [\![a^{\mathbb{P}}_i]\!]^{\mathbb{P}} = [\ x^i_1, \cdots, x^i_k\ ]$$

$$[\![a^{\mathbb{P}}_1 == a^{\mathbb{P}}_2]\!]^{\mathbb{P}}_{\sigma,\sigma^{\mathbb{P}}} := x^1_1 == x^2_1 \wedge \cdots \wedge x^1_k == x^2_k \text{ where } [\![a^{\mathbb{P}}_i]\!]^{\mathbb{P}} = [\ x^i_1, \cdots, x^i_k\ ]$$

$$[\![c]\!]^{\mathbb{P}}_{\sigma,\sigma^{\mathbb{P}}} := c \qquad [\![v]\!]^{\mathbb{P}}_{\sigma,\sigma^{\mathbb{P}}} := \sigma(v) \qquad [\![a_1\ op_a\ a_2]\!]^{\mathbb{P}}_{\sigma,\sigma^{\mathbb{P}}} := [\![a_1]\!]^{\mathbb{P}}_{\sigma,\sigma^{\mathbb{P}}}\ op_a\ [\![a_2]\!]^{\mathbb{P}}_{\sigma,\sigma^{\mathbb{P}}}$$

$$[\![v = a]\!]^{\mathbb{P}}_{\sigma,\sigma^{\mathbb{P}}} := (\sigma[v \hookleftarrow [\![a]\!]^{\mathbb{P}}_{\sigma,\sigma^{\mathbb{P}}}], \sigma^{\mathbb{P}}) \qquad [\![v^{\mathbb{P}} = a^{\mathbb{P}}]\!]^{\mathbb{P}}_{\sigma,\sigma^{\mathbb{P}}} := (\sigma, \sigma^{\mathbb{P}}[v^{\mathbb{P}} \hookleftarrow [\![a^{\mathbb{P}}]\!]^{\mathbb{P}}_{\sigma,\sigma^{\mathbb{P}}}])$$

Fig. 3: Modular semantics.

prime numbers. The structure of an `IMP-MOD` program is similar to an `IMP` program, but `IMP-MOD` supports two types of variables and arithmetic expressions: the regular `IMP` ones (i.e., $v$, $a$, and $b$), which operate over an integer semantics, and the modular ones (i.e., $v^{\mathbb{P}}$, $a^{\mathbb{P}}$, and $b^{\mathbb{P}}$), which take as an additional parameter the set of primes $\mathbb{P}$ and operate over a modular semantics. The semantics of some of the key constructs of `IMP-MOD` is shown in Figure 3.

The key idea of the modular semantics is that the value of each program variable in $v^{\mathbb{P}}$ and arithmetic expressions in $a^{\mathbb{P}}$ is denoted by a tuple of values, one for each prime number $p_i \in \mathbb{P}$. For example, the value of the constant $c^{\mathbb{P}}$ is represented by the tuple $[c \bmod p_1, \cdots, c \bmod p_k]$, where each individual value denotes the remainder of $c$ when divided by the prime number $p_i \in \mathbb{P}$. Formally, the program $f$ has two sets of variables $V^{\mathbb{Z}} = \{v_1, \cdots, v_n\}$ and $V^{\mathbb{P}} = \{v^{\mathbb{P}}_1, \cdots, v^{\mathbb{P}}_m\}$, which contain all the integer and prime variables respectively, and a set of holes $H = \{??_1, \ldots, ??_k\}$. The denotation function, uses two valuation functions: $(i)$ $\sigma : V^{\mathbb{Z}} \cup H \to \mathbb{Z}$, which maps variables and holes to integer values, $(ii)$ $\sigma^{\mathbb{P}} : V^{\mathbb{P}} \to [0, p_1) \times \cdots \times [0, p_k)$, which maps primed variables to modular values. The expression $\textsc{toPrime}(a)$ converts the integer value of an integer expression $a$ to a modular tuple. Arithmetic expressions in $a^{\mathbb{P}}$ are computed using modular values with the result being obtained using modular arithmetic with respect to the corresponding primes in $\mathbb{P}$. Note that the only comparison operator allowed over modular expressions is $==$ and that the division operator *cannot* be applied to modular expressions. While the syntax does not directly allow for holes to be represented modularly—i.e., we do not have expressions of the form $??^{\mathbb{P}}$—an expression of the form $\textsc{toPrime}(??)$ effectively achieves the objective of representing a hole $??$ modularly.

#### 4.3 Equivalence between the two Semantics

Next, we provide an alternative integer semantics, which applies the `IMP` integer semantics to modular expressions and show that, under some assumptions on the values manipulated by the program, the modular and integer semantics are equivalent. We will use this result to build our modified synthesis algorithm.

$$[\![\textsc{toPrime}(a)]\!]_{\sigma_1,\sigma_2} := [\![a]\!]_{\sigma_1,\sigma_2} \qquad [\![v^{\mathbb{P}}]\!]_{\sigma_1,\sigma_2} := \sigma_2(v^{\mathbb{P}}) \qquad [\![c^{\mathbb{P}}]\!]_{\sigma_1,\sigma_2} := c$$

$$[\![a_1^{\mathbb{P}}\ op_a^{\mathbb{P}}\ a_2^{\mathbb{P}}]\!]_{\sigma_1,\sigma_2} := [\![a_1^{\mathbb{P}}]\!]_{\sigma_1,\sigma_2}\ op_a\ [\![a_2^{\mathbb{P}}]\!]_{\sigma_1,\sigma_2} \qquad [\![a_1^{\mathbb{P}}{==}a_2^{\mathbb{P}}]\!]_{\sigma_1,\sigma_2} := [\![a_1^{\mathbb{P}}]\!]_{\sigma_1,\sigma_2}{==}[\![a_2^{\mathbb{P}}]\!]_{\sigma_1,\sigma_2}$$

$$[\![c]\!]_{\sigma_1,\sigma_2} := c \qquad [\![v]\!]_{\sigma_1,\sigma_2} := \sigma_1(v) \qquad [\![a_1\ op_a\ a_2]\!]_{\sigma_1,\sigma_2} := [\![a_1]\!]_{\sigma_1,\sigma_2}\ op_a\ [\![a_2]\!]_{\sigma_1,\sigma_2}$$

$$[\![v = a]\!]_{\sigma_1,\sigma_2} := (\sigma_1[v \hookleftarrow [\![a]\!]_{\sigma_1,\sigma_2}], \sigma_2) \qquad [\![v^{\mathbb{P}} = a^{\mathbb{P}}]\!]_{\sigma_1,\sigma_2} := (\sigma_1, \sigma_2[v^{\mathbb{P}} \hookleftarrow [\![a^{\mathbb{P}}]\!]_{\sigma_1,\sigma_2}])$$

Fig. 4: Integer semantics.

*Integer Semantics* The *integer semantics* of `IMP-MOD` is shown in Figure 4 (denoted $[\![\cdot]\!]_{\sigma_1,\sigma_2}$). In this semantics, modular expressions are evaluated as integer expressions using the same semantics as for `IMP`—i.e., the values of modular variables and modular arithmetic expressions are denoted by integer values. Therefore, in the integer semantics, we use two valuation functions $\sigma_1 : V^{\mathbb{Z}} \cup H \mapsto \mathbb{Z}$ mapping variables and holes to integers and $\sigma_2 : V^{\mathbb{P}} \mapsto \mathbb{Z}$ mapping modular variables to integers.

*Relation between the Two Semantics* We now show that the modular semantics is, in some sense, equivalent to the integer semantics. For the rest of this section, we fix a set of distinct primes $\mathbb{P} = \{p_1, \cdots, p_k\}$.

To prove the equivalence of the two program semantics, we will require the values of modular expressions to lie in some range that is covered by the prime numbers in $\mathbb{P}$. The following definition captures this restriction.

**Definition 1.** *Given a modular arithmetic expression $a^{\mathbb{P}}$ (resp. Boolean expression $b$) and some integers $L < U$, we say $a^{\mathbb{P}}$ with context $(\sigma_1, \sigma_2)$ is* uniformly in the range *$R := [L, U)$ —$a^{\mathbb{P}} \in_{\sigma_1,\sigma_2} R$ for short—if under the integer semantics, all evaluation of modular subexpressions of $a^{\mathbb{P}}$ (resp. $b$) are in the range $R$:*

- *$a^{\mathbb{P}} \in_{\sigma_1,\sigma_2} R$, iff $[\![a^{\mathbb{P}}]\!]_{\sigma_1,\sigma_2} \in R$;*
- *$a_1^{\mathbb{P}}\ {==}\ a_2^{\mathbb{P}} \in_{\sigma_1,\sigma_2} R$, iff $a_1^{\mathbb{P}} \in_{\sigma_1,\sigma_2} R$, $a_2^{\mathbb{P}} \in_{\sigma_1,\sigma_2} R$;*
- *$b_1$ `and` $b_2 \in_{\sigma_1,\sigma_2} R$, iff $b_1 \in_{\sigma_1,\sigma_2} R$, $b_2 \in_{\sigma_1,\sigma_2} R$;*
- *$b_1$ `or` $b_2 \in_{\sigma_1,\sigma_2} R$, iff $b_1 \in_{\sigma_1,\sigma_2} R$, $b_2 \in_{\sigma_1,\sigma_2} R$;*
- *`not` $b \in_{\sigma_1,\sigma_2} R$, iff $b \in_{\sigma_1,\sigma_2} R$;*
- *$a_1\ op_c\ a_2 \in_{\sigma_1,\sigma_2} R$ for any arithmetic expressions $a_1$, $a_2$ and operator $op_c$.*

Given a valuation function $\sigma : V^{\mathbb{P}} \mapsto \mathbb{Z}$, we write $m_{\mathbb{P}} \circ \sigma$ to denote the modular valuation obtained by applying the $m_{\mathbb{P}}$ function to $\sigma$—i.e., for every $v^{\mathbb{P}} \in V^{\mathbb{P}}$, $(m_{\mathbb{P}} \circ \sigma)(v^{\mathbb{P}}) = m_{\mathbb{P}}(\sigma(v^{\mathbb{P}}))$. Similarly, for a modular valuation function $\sigma^{\mathbb{P}} : V^{\mathbb{P}} \to [0, p_1) \times \cdots [0, p_k)$, we denote $m_{\mathbb{P}}^{-1,R} \circ \sigma^{\mathbb{P}}$ the integer valuation from $V^{\mathbb{P}}$ to $R$ such that, for every $v^{\mathbb{P}} \in V^{\mathbb{P}}$, $(m_{\mathbb{P}}^{-1,R} \circ \sigma^{\mathbb{P}})(v^{\mathbb{P}}) = m_{\mathbb{P}}^{-1,R}(\sigma^{\mathbb{P}}(v^{\mathbb{P}}))$. The following lemma shows that, when the values of modular arithmetic expressions lay in an interval of size $N = p_1 \cdot \ldots \cdot p_k$ the modular and integer semantics of modular arithmetic expressions are equivalent.

**Lemma 2.** *Given a set of primes* $\mathbb{P} = \{p_1, \cdots, p_k\}$*, an arithmetic expression* $a^{\mathbb{P}}$*, and two valuation functions* $\sigma_1 : V^{\mathbb{Z}} \cup H \mapsto \mathbb{Z}$ *and* $\sigma_2 : V^{\mathbb{P}} \mapsto \mathbb{Z}$*, we have*

$$m_{\mathbb{P}}([\![a^{\mathbb{P}}]\!]_{\sigma_1,\sigma_2}) = [\![a^{\mathbb{P}}]\!]^{\mathbb{P}}_{\sigma_1, m_{\mathbb{P}}\circ\sigma_2}$$

*Moreover, if there exists an interval* $R$ *of size* $N = p_1 \cdot \ldots \cdot p_k$ *such that* $a^{\mathbb{P}} \in_{\sigma_1,\sigma_2} R$*, then*

$$m_{\mathbb{P}}^{-1,R}([\![a^{\mathbb{P}}]\!]^{\mathbb{P}}_{\sigma_1, m_{\mathbb{P}}\circ\sigma_2}) = [\![a^{\mathbb{P}}]\!]_{\sigma_1,\sigma_2}.$$

Similarly, we show that the two semantics are also equivalent for Boolean expressions.

**Lemma 3.** *Given a set of primes* $\mathbb{P} = \{p_1, \cdots, p_k\}$*, an interval* $R$ *of size* $N = p_1 \cdot \ldots \cdot p_k$*, a Boolean expression* $b$*, and two valuation functions* $\sigma_1 : V^{\mathbb{Z}} \cup H \mapsto \mathbb{Z}$ *and* $\sigma_2 : V^{\mathbb{P}} \mapsto \mathbb{Z}$*, if* $b \in_{\sigma_1,\sigma_2} R$*, then* $[\![b]\!]_{\sigma_1,\sigma_2} = [\![b]\!]^{\mathbb{P}}_{\sigma_1, m_{\mathbb{P}}\circ\sigma_2}$.

We are now ready to show the equivalence between the modular semantics and the integer semantics for programs $P \in$ `IMP-MOD`. The semantics of a program $P = f(V^{\mathbb{Z}}, V^{\mathbb{P}}, H)\ \{s\}$ is a map from valuations to valuations, i.e., given a valuation $\sigma_1 : V^{\mathbb{Z}} \to \mathbb{Z}$ for integer variables, a valuation $\sigma_2 : V^{\mathbb{P}} \to \mathbb{Z}$ for modular variables and a valuation $\sigma^H : H \to \mathbb{Z}$ for holes, we have $[\![P]\!](\sigma_1, \sigma_2, \sigma^H) = [\![s]\!]_{\sigma_1 \cup \sigma^H, \sigma_2}$ and $[\![P]\!]^{\mathbb{P}}(\sigma_1, \sigma_2, \sigma^H) = [\![s]\!]^{\mathbb{P}}_{\sigma_1 \cup \sigma^H, m_{\mathbb{P}}\circ\sigma_2}$. Therefore, it is sufficient to show that the two semantics are equivalent for any statement $s$.

The two semantics are equivalent for a statement $s$ if, under the same input valuations, the resulting valuations of the semantics can be translated to each other. Formally, given valuations $\sigma_1$, $\sigma_2$ and an interval $R$ of size $N$, we say $[\![s]\!]_{\sigma_1,\sigma_2} \equiv_{\mathbb{P}} [\![s]\!]^{\mathbb{P}}_{\sigma_1, m_{\mathbb{P}}\circ\sigma_2}$ iff $\sigma_1' = \sigma_1''$, $m_{\mathbb{P}} \circ \sigma_2' = \sigma_2^{\mathbb{P}}$ and $\sigma_2' = m_{\mathbb{P}}^{-1,R} \circ \sigma_2^{\mathbb{P}}$ where $[\![s]\!]_{\sigma_1,\sigma_2} = (\sigma_1', \sigma_2')$ and $[\![s]\!]^{\mathbb{P}}_{\sigma_1, m_{\mathbb{P}}\circ\sigma_2} = (\sigma_1'', \sigma_2^{\mathbb{P}})$.

We define uniform inclusion for statements.

**Definition 2.** *Given a set of primes* $\mathbb{P}$*, two integers* $L < U$ *and a statement* $s$*, we say* $s$ *with context* $(\sigma_1, \sigma_2)$ *is* uniformly in the range $R := [L, U)$*—*$s \in_{\sigma_1,\sigma_2} R$ *for short—if under the integer semantics, all evaluation of modular subexpressions of* $s$ *are in the range* $R$*:*

- $(v^{\mathbb{P}} = a^{\mathbb{P}}) \in_{\sigma_1,\sigma_2} R$ *iff* $a^{\mathbb{P}} \in_{\sigma_1,\sigma_2} R$.
- *while*$(b)\{s\} \in_{\sigma_1,\sigma_2} R$ *iff* $s \in_{\sigma_1,\sigma_2} R$ *and* $b \in_{\sigma_1,\sigma_2} R$.
- $s_1; s_2 \in_{\sigma_1,\sigma_2} R$ *iff* $s_1 \in_{\sigma_1,\sigma_2} R$ *and* $s_2 \in_{\sigma_1,\sigma_2} R$.
- *if*$(b)$ $s_1$ *else* $s_2 \in_{\sigma_1,\sigma_2} R$ *iff* $s_1 \in_{\sigma_1,\sigma_2} R$, $s_2 \in_{\sigma_1,\sigma_2} R$ *and* $b \in_{\sigma_1,\sigma_2} R$.
- *assert* $b \in_{\sigma_1,\sigma_2} R$ *iff* $b \in_{\sigma_1,\sigma_2} R$.

At last, the two semantics are equivalent for statements.

**Theorem 2.** *Given a set of primes* $\mathbb{P} = [p_1, \cdots, p_k]$*, a statement* $s$ *and two valuation functions* $\sigma_1 : V^{\mathbb{Z}} \cup H \to \mathbb{Z}$ *and* $\sigma_2 : V^{\mathbb{P}} \to \mathbb{Z}$*, if there exists an interval* $R$ *of size* $N$ *such that* $s \in_{\sigma_1,\sigma_2} R$*, then* $[\![s]\!]_{\sigma_1,\sigma_2} \equiv_{\mathbb{P}} [\![s]\!]^{\mathbb{P}}_{\sigma_1, m_{\mathbb{P}}\circ\sigma_2}$.

**Algorithm 1:** returns variables that should be tracked using modular/integer semantics.

```
/* f: sketched function, V^P variables to be tracked modularly, V^Z
   variables to be tracked with integer values                    */
function DataFlowAnalysis(f)
    S ← {/, <, >, ≤, ≥}; V^Z ← ∅
    for op ∈ S do
        /* Compute all variables v that may flow into op           */
        V^Z ← V^Z ∪ Dataflow(op, f)
    V^P ← V \ V^Z
    return (V^Z, V^P)
```

## 5 From IMP to IMP-MOD Programs

In this section, we develop a data flow analysis for detecting variables in IMP programs for which it is sound to track values modularly. We then use this data flow analysis to rewrite an IMP program to an equivalent IMP-MOD program.

### 5.1 Data Flow Analysis

The formalization of IMP-MOD in Section 4.2 made it clear that the modular semantics is only appropriate when integer values are manipulated using addition, multiplication, subtraction, and equality. Other operations like division and less-than comparison cannot be computed soundly in modular arithmetic.

*Example 4.* Consider an integer variable $x$ with modular value $x_2$ under modulus 2 and $x_3$ under modulus 3, and an integer variable $y$ with modular value $y_2$, $y_3$ under corresponding moduli. Then the assignment of $x = y + y$; implies $x_2 = (y_2 + y_2) \mod 2$; and $x_3 = (y_3 + y_3) \mod 3$. However, $x = x/y$; does not imply $x_2 = (x_2/y_2) \mod 2$; and $x_3 = (x_3/y_3) \mod 3$.

We now define a data flow analysis (shown in Algorithm 1) for computing which variables in a program must be tracked with the integer semantics (i.e., the set $V^{\mathbb{Z}}$) and which variables can be soundly tracked using the modular semantics (i.e., the set $V^{\mathbb{P}}$). For each operator *op* in $\{/, <, >, \leq, \geq\}$, the analysis computes the set of variables that *may* flow into the operands of an expression of the form $e_1$ *op* $e_2$. In practice, this is done via *backward may analysis*, noted as `Dataflow` procedure in Algorithm 1. The obtained set of variables must be tracked using the integer semantics. The remaining variables will never flow into a problematic operator and can therefore be tracked using the modular semantics.

*Implementation Remark* Since our implementation also supports arrays and recursion, the data flow analysis in Algorithm 1 is inter-procedural and the set $S$ also contains the array indexing operator [ ]—i.e., given an expression $arr[a]$, if a variable $v$ may flow into $a$, then $a$ must be tracked using the integer semantics.

$$\mathrm{R}_a(a) = \begin{cases} v^{\mathbb{P}} & \text{if } a \equiv v \text{ and } v \in V^{\mathbb{P}} \\ c^{\mathbb{P}} & \text{if } a \equiv c \\ \mathrm{R}_a(a_1)\ op_a^{\mathbb{P}}\ \mathrm{R}_a(a_2) & \text{if } a \equiv a_1\ op_a^{\mathbb{P}}\ a_2 \\ \textsc{toPrime}(a) & \text{otherwise} \end{cases}$$

$$\mathrm{R}_b(b) = \begin{cases} \mathrm{R}_a(a_1)\ \texttt{==}\ \mathrm{R}_a(a_2) & \text{if } b \equiv a_1\ \texttt{==}\ a_2 \\ \mathrm{R}_b(b_1)\ \texttt{and}\ \mathrm{R}_b(b_2) & \text{if } b \equiv b_1\ \texttt{and}\ b_2 \\ \texttt{not}\ \mathrm{R}_b(b_1) & \text{if } b \equiv \texttt{not}\ b_2 \\ b & \text{otherwise} \end{cases}$$

$$\mathrm{R}_s(s) = \begin{cases} \mathrm{R}_s(s_1); \mathrm{R}_s(s_2) & \text{if } s \equiv s_1; s_2 \\ v\ =\ a & \text{if } s \equiv v = a \text{ and } v \in V^{\mathbb{Z}} \\ v^{\mathbb{P}}\ =\ \mathrm{R}_a(a) & \text{if } s \equiv v = a \text{ and } v \in V^{\mathbb{P}} \\ \texttt{if}(\mathrm{R}_b(b))\ \mathrm{R}_s(s_0)\ \texttt{else}\ \mathrm{R}_s(s_1) & \text{if } s \equiv \texttt{if}(b)\ s_0\ \texttt{else}\ s_1 \\ \texttt{while}(\mathrm{R}_b(b))\ \{\mathrm{R}_s(s)\} & \text{if } s \equiv \texttt{while}\ b\ \{s\} \\ \texttt{assert}\ \mathrm{R}_b(b) & \text{if } s \equiv \texttt{assert}\ b \end{cases}$$

Fig. 5: Subset of rules for the translation from IMP to IMP-MOD programs. Rules are parametric in $V^{\mathbb{Z}}$, $V^{\mathbb{P}}$ with $\mathbb{P}$: $\mathrm{R}_f(f(V, ??)\{s\}) = f(V^{\mathbb{Z}}, V^{\mathbb{P}}, ??)\{\mathrm{R}_s(s)\}$.

Furthermore, while in our formalization we allow variables to be tracked using only one of the two semantics, in our implementation, we allow variables to be tracked differently (using actual values or modular values) at different program points by tracking, for each variable $v$, the program points for which the actual value of $v$ is needed, which is done by using the same data-flow analysis. In this case, a variable might initially need to be tracked using actual values but can later be tracked using modular values.

*Example 5.* Consider the sketch program `polyArray` in Figure 1b. For this program, Algorithm 1 will return that the variables `x` and `y` can be tracked modularly. However, the variables `i` and `n` must be tracked using the integer semantics since they are used in a `<` operation and as array indices.

#### 5.2 From IMP to IMP-MOD

Now that we have computed what sets of variables can be tracked modularly, we can transform the IMP program into an IMP-MOD program. The transformation $\mathrm{R}_f$ that rewrites $f$ into an IMP-MOD program is shown in Figure 5. The key idea of the program transformation is to use the sets $V^{\mathbb{Z}}$ and $V^{\mathbb{P}}$ to only rewrite variables and sub-expressions of $f$ for which the modular arithmetic can be performed soundly.

Once we get a solution for the IMP-MOD program as hole values, we can get a solution for the IMP program by mapping the hole to integer values given by the integer semantics.

*Example 6.* Consider a program where the dataflow analysis computes $V^{\mathbb{Z}} = \{i, n\}$ and $V^{\mathbb{P}} = \{x\}$. The statement $x = x + i + 1$ is rewritten to $x^{\mathbb{P}} = x^{\mathbb{P}} + \text{TOPRIME}(i) + 1^{\mathbb{P}}$.

The transformation $\text{R}_f$ is sound.

**Theorem 3.** *Given an* `IMP` *program* $f$*, and sets* $V^{\mathbb{Z}}$ *and* $V^{\mathbb{P}}$ *resulting from the data flow analysis on* $f$*, the program* $\text{R}_f(f)$ *is in the* `IMP-MOD` *language. Moreover,* $[\![f]\!]^{\mathit{IMP}} = [\![\text{R}_f(f)]\!]$.

## 6 Solving IMP-MOD Sketches

In this section, we discuss how synthesis in the modular semantics relates to synthesis in the integer semantics and provide an incremental algorithm for solving `IMP-MOD` sketches.

### 6.1 Synthesis in IMP-MOD

Given a set of integers $R$ we say that a variable valuation $\sigma$ is in $R$ (denoted $\sigma \in R$) if for every $v$, we have $\sigma(v) \in R$. Similarly to what we saw in Section 3, we assume that the sketch has to be solved for finite ranges of possible values for the hole ($R_H$) and input values ($R_{in}$). Solving an `IMP-MOD` problem $P = f(V, V^{\mathbb{P}}, H)\{s\}$ for the integer semantics amounts to solving the following constraint:

$$\exists \sigma^H \in R_H . \forall \sigma_1, \sigma_2 \in R_{in} . [\![s]\!]_{\sigma_1 \cup \sigma^H, \sigma_2} \neq \bot.$$

According to Theorem. 2, given a set of distinct primes $\mathbb{P} = \{p_1, \cdots, p_k\}$ and variable valuations $\sigma^H, \sigma_1$, and $\sigma_2$, if there exists a range $R$ of size $N = p_1 \cdot \ldots \cdot p_k$ such that $s \in_{\sigma_1 \cup \sigma^H, \sigma_2} R$, the modular semantics and the integer semantics are equivalent to each other. Using this observation, we can define the set of variable valuations for which the two semantics are guaranteed to be equivalent:

$$\mathcal{I}_R^{\mathbb{P}} := \left\{ (\sigma_1, \sigma_2) \mid \forall \sigma^H \in R_H . \exists R .\ |R| = N \wedge s \in_{\sigma_1 \cup \sigma^H, \sigma_2} R \right\}.$$

Since for every $\sigma^H \in R_H$ and $\sigma_1, \sigma_2 \in \mathcal{I}_R^{\mathbb{P}}$ we have that $[\![s]\!]^{\mathbb{P}}_{\sigma_1 \cup \sigma^H, m_{\mathbb{P}} \circ \sigma_2} = [\![s]\!]_{\sigma_1 \cup \sigma^H, \sigma_2}$, any solution to an `IMP-MOD` program in the modular semantics is also a solution to the following formula in the integer semantics:

$$\exists \sigma^H \in R_H . \forall \sigma_1, \sigma_2 \in \mathcal{I}_R^{\mathbb{P}} . [\![s]\!]_{\sigma_1 \cup \sigma^H, \sigma_2} \neq \bot.$$

When all valuations in $\sigma_1, \sigma_2 \in R_{in}$ are also elements of $\mathcal{I}_R^{\mathbb{P}}$, any solution to an `IMP-MOD` program in the modular semantics is guaranteed to be a correct solution under the integer semantics.

To summarize, if the synthesizer returns UNSAT for the `IMP-MOD` program, the problem is unrealizable and does not admit a solution. When it returns a solution, the solution is correct if it only produces valuations in the range allowed by

**Algorithm 2:** Incremental synthesis for `IMP-MOD`.

/* $f$: function, $\mathbb{P}$: set of primes */

**function** `IncrementalSynthesis`$(f, \mathbb{P})$

$\mathbb{P}' \leftarrow [p_1]$

$f_{\mathsf{syn}} \leftarrow$ `Synthesis`$(f, \mathbb{P}')$

**while** $\exists p_{cex} \in \mathbb{P}: \neg$ *Verify*$(f_{syn}, p_{cex})$ **do**

$\mathbb{P}' \leftarrow \mathbb{P}' \cup p_{\mathsf{cex}}$

$f_{\mathsf{syn}} \leftarrow$ `Synthesis`$(f, \mathbb{P}')$

**if** $f_{syn} ==$ *UNSAT* **then return** $\emptyset$ ;

**return** $f_{syn}$

the choice of prime numbers. In practice, one can use a verifier to check the correctness of the synthesized solution and add more prime numbers to the modular synthesizer if needed. In fact, this is the main idea behind the counterexample-guided inductive synthesis algorithm used by SKETCH (Section 3).

#### 6.2 Incremental Synthesis Algorithm

In this section, we propose an incremental synthesis algorithm that builds on the following observation. The set of variable valuations for which modular and integer semantics are equivalent increases monotonically in the size of $\mathbb{P}$:

$$\mathbb{P}_1 \subseteq \mathbb{P}_2 \Longrightarrow \mathcal{I}_R^{\mathbb{P}_1} \subseteq \mathcal{I}_R^{\mathbb{P}_2}. \tag{1}$$

Algorithm 2 uses Equation 1 to add prime numbers lazily during the synthesis process. The algorithm first constructs a set $\mathbb{P}' = \{p_1\}$ with the first prime number $p_1 \in \mathbb{P}$ and synthesizes a solution that is correct for computations modulo the set $\mathbb{P}'$. It then checks if the synthesized solution $f_{\mathsf{syn}}$ satisfies the assertions with respect to all prime numbers in $\mathbb{P}$. If yes, $f_{\mathsf{syn}}$ is returned as the solution. Otherwise, the algorithm finds a prime $p_{\mathsf{cex}} \in \mathbb{P}$ where `Verify`$(f_{\mathsf{syn}}, p_{\mathsf{cex}})$ does not hold and it adds it to the set $\mathbb{P}'$ continuing the iterative algorithm. Due to Equation 1, Algorithm 2 is sound and complete with respect to the synthesis algorithm that considers the full prime set $\mathbb{P}$ all at once.

In practice, the user could use domain knowledge to estimate a suitable set of primes or alternatively use our incremental algorithm to discover appropriate prime sets. The set of prime numbers $\{2, 3, 5, 7, 11, 13, 17\}$ could usually instantiate a range $R$ that is large enough for most synthesis tasks based on SKETCH.

### 7 Complexity of Rewritten Programs

In this section, we analyze how many bits are necessary to encode numbers for both semantics using unary and binary bit-vector encodings of integers (Sec. 7.1 and 7.2), and show how many prime numbers are necessary in the modular semantics to cover values up to a certain bound (Sec. 7.3). The following results build upon several number theory results that the reader can consult at [9, 15].

#### 7.1 Bit-complexity of Binary Encoding

In this section, we analyze how many bits are necessary when representing an interval of size $N$ in binary in our modular semantics. In the rest of the section, we consider the set of primes $\mathbb{P}_n = \{p \mid p < n\} = \{p_1, \ldots, p_k\}$ containing the prime numbers that have value smaller than $n$. We will show in Section 8 that this choice of prime number also yields good performance in practice. Concretely, we are interested in knowing what is the magnitude of the number $N = p_1 \cdot \ldots \cdot p_k$ and how many bits are used to represent the numbers in $\mathbb{P}_n$.

We start by introducing the notion of primorial.

**Definition 3 (Primorial).** *Given a number $n$, the* primorial $n\#$ *is defined as the product of all primes smaller than $n$—i.e.,* $n\# = \prod_{p \in \mathbb{P}_n} p$.

The primorial captures the size $N$ of the interval covered by the Chinese Remainder Theorem when using prime numbers up to $n$. The following number theory result gives us a close form for the primorial and shows that the number $N$ has approximately $n$ bits.

$$n\# = e^{(1+o(1))n} = 2^{(1+o(1))n} \tag{2}$$

We use another number theory notion to quantify the number of bits in $\mathbb{P}_n$.

**Definition 4 (Chebyshev function).** *Given a number $n$, the* Chebyshev function $\vartheta(n)$ *is the sum of the logarithms of all the prime numbers smaller than $n$—i.e.,* $\vartheta(n) = \sum_{p \in \mathbb{P}_n} \log p$.

The following number theory result relates the primorial to the Chebyshev function.

$$\vartheta(n) = \log(n\#) = \log 2^{(1+o(1))n} = (1 + o(1))n \tag{3}$$

Aside from rounding errors, the Chebyshev function captures the number of bits required to represent the numbers in $\mathbb{P}_n$. To obtain a more precise bound on this number, we need a bound for the formula $\sum_{p \in \mathbb{P}_n} \lceil \log p \rceil$.

We start by recalling the following fundamental number theory result.

**Theorem 4 (Prime number theorem).** *The set $\mathbb{P}_n$ has size approximately $n/\log n$.*

Using Theorem 4, we get the following result.

$$\sum_{p \in \mathbb{P}_n} \lceil \log p \rceil \leq n/\log n + \sum_{p \in \mathbb{P}_n} \log p \approx (1 + o(1))n \tag{4}$$

Representing a number $e^n$ in a classic binary encoding requires $\log_2(e^n) = (1 + o(1))n$ bits and, combining Equations 2 and 4, we get the following result.

**Theorem 5.** *Representing a number $2^n$ in binary requires $(1+o(1))n$ bits under both modular and integer semantics.*

Hence, representing a number in binary requires the same number of bits in the both semantics.

*Example 7.* Consider the set $\mathbb{P}_{18} = \{2, 3, 5, 7, 11, 13, 17\}$, which can model an interval of $N = 510,510$ integers (i.e., $n = 18$ in Theorem 5). Representing $N$ in binary requires 19 bits while the binary representations of all the primes in $\mathbb{P}_{18}$ use 22 bits. Both numbers are close to 18 as predicted by the theorem.

#### 7.2 Bit-complexity of Unary Encoding

As discussed in Sec. 3, the default SKETCH solver encodes numbers using a unary encoding—i.e., SKETCH requires $2^n$ bits to encode the number $2^n$. Representing the same number in unary under the modular semantics requires only prime numbers smaller than $n$ and therefore $\sum_{p \in \mathbb{P}_n} p$ bits. We can then use the following closed form to approximate this quantity.

$$\sum_{p \in \mathbb{P}_n} p \sim \frac{n^2}{2 \log n} \tag{5}$$

Equation 5 yields the following theorem.

**Theorem 6.** *Representing a number $2^n$ in unary requires $2^n$ bits in the integer semantics and approximately $\frac{n^2}{2 \log n}$ bits in the modular semantics.*

These results show that, under a unary encoding, the modular semantics is exponentially more succinct than the integer semantics.

*Example 8.* Consider again the prime set $\mathbb{P}_{18} = \{2, 3, 5, 7, 11, 13, 17\}$, which can model an interval of $N = 510,510$ integers. Representing $N$ in unary requires 510,510 bits. On the other hand, the sum of the bits in the unary encoding of the primes in $\mathbb{P}_{18}$ is 58.

#### 7.3 Number of Required Primes

We analyze how many primes are needed to represent a certain number in the modular semantics. We start by introducing the following alternative version of the primorial.

**Definition 5 (Prime Primorial).** *For the $n$-th prime number $p_n$, the* prime primorial *$p_n\#$ is defined as the product of the first $n$ primes—i.e., $p_n\# = \prod_{k=1}^{n} p_i$.*

The following known number theory result gives us an approximation for the prime primorial.

$$p_n\# = e^{(1+o(1))n \log n} \tag{6}$$

Notice how the approximation of the primorial differs from that of the prime primorial. This is due to the fact that prime numbers are sparse—i.e., the $n$-th prime number is approximately $n \log n$.

Using Equation 6 we obtain the following result.

**Theorem 7.** *Representing numbers in an interval of size $N = e^{n \log n}$ in the modular semantics requires the first n prime numbers.*

Since the relation $k = n \log n$ does not admit a closed form for $n$, we cannot derive exactly how many primes are needed to represent a number $2^k$ with $k$ bits. It is however clear from the theorem that the number of required primes grows slower than $k$.

### 8 Evaluation

We implemented a prototype of our technique as a simple compiler in Java. Our implementation provides a simplified SKETCH frontend, which only allows the limited syntax we support. Given a SKETCH file, our tool rewrites it into a different SKETCH file that operates according to the modular semantics. We will use UNARY to denote the result obtained by running the default version of SKETCH with unary integer encoding on the original SKETCH file, BINARY to denote the result obtained by running the version of SKETCH using an SMT-like native integer solver based on binary integer encoding, UNARY-P to denote the result of running the default SKETCH version on our modified SKETCH file, and UNARY-P-INC to denote the result of running the default version of SKETCH on the file generated by the incremental version of our algorithm described in Section 6. As expected from our theory, the prime technique is not beneficial for the SMT-like native integer solver and always results in worse runtime. Therefore, we do not present data for this solver. All experiments were performed on a machine with 4.0GHz Intel Core i7 CPU with 16GB RAM with SKETCH-1.7.5 and we use a timeout value of 300 seconds (we also report out-of-memory errors as timeouts).

Our evaluation answers the following research questions:

**Q1** How does the performance of UNARY-P compare to UNARY and BINARY?
**Q2** How does the incremental algorithm compare to the non-incremental one?
**Q3** Is UNARY-P's performance sensitive to the set of selected prime numbers?
**Q4** How many primes are needed by UNARY-P to produce correct solutions?
**Q5** Does UNARY generate larger SAT queries than UNARY-P?

#### 8.1 Benchmarks

We perform our evaluation on three families of programs.

*Polynomials* The first set of benchmarks contains 81 variants of the polynomial synthesis problem presented in Figure 1. The original version of this benchmark appears in the SKETCH benchmark suite under the name `polynomial.sk`. For each benchmark, we generate a random polynomial $f$, random inputs $\{\overrightarrow{x}\}$, and take the set $\{(\overrightarrow{x}, f(x))\}$ as specification. Each benchmark in this set has the following parameters: `#Ex`$\in \{2, 4, 6\}$ is the number of input-output examples as specification, `cbits`$\in \{5, 6, 7\}$ denote the number of bits hole values can use, `exIn`$\in \{[-10, 10], [-30, 30], [-50, 50]\}$ denotes the range of randomly generated input examples and `coeff`$\in \{[-10, 10], [-30, 30], [-50, 50]\}$ denotes the range of randomly generated coefficients in the polynomial $f$.

*Invariants* The second set of benchmarks contain 46 variants of two invariant generation problems obtained from a public set of programs that require polynomial invariants to be verified [8]. We selected the two programs in which at least one variable could be tracked modularly by our tool (the other programs involved complex array operations or inequality operators) and turned the verification problems into synthesis problems by asking SKETCH to find a polynomial equality (using the program variables) that is an invariant for the loop in the program. To control the size of the magnitudes of the inputs, we only require the invariants to hold for a fixed set of input examples.

The first problem, `mannadiv`, iteratively computes the remainder and the quotient of two numbers given as input. The invariant required to verify `mannadiv` is a polynomial equality of degree 2 involving 5 variables. The SKETCH template required to describe the space of all polynomial equalities has 32 holes and cannot be handled by any of the SKETCH solvers we consider. We therefore simplify the invariant synthesis problems in two ways. In the first variant, we reduce the ranges of the hole values in the templates by considering `cbits` $\in \{2, 3\}$. In the second variant, we set `cbits` $= \{5, 6, 7\}$, but reduce the number of missing hole values to 4 (i.e., we provide part of the invariant). Each benchmark takes two random inputs and we consider the following input ranges $\{[1, 50], [1, 100]\}$. In total, we have 10 benchmarks for `mannadiv`.

The second problem, `petter`, iteratively computes the sum $\sum_{1\leq i\leq n} i^5$ for a given input $n$. The invariant required to verify `petter` is a polynomial equality of degree 6 involving 3 variables. The SKETCH template required to describe all such polynomial equalities has 56 holes and cannot be handled by any of the SKETCH solvers we consider. We consider the following simplified variants of the problem: ($i$) `petter_0` computes $\sum_{1\leq i\leq n} 1$ and requires a polynomial invariant of degree one, ($ii$) `petter_x` computes $\sum_{1\leq i\leq n} x$ for a given input variable $x$ and requires a polynomial invariant of degree two, ($iii$) `petter_1` computes $\sum_{1\leq i\leq n} i$ and requires a polynomial invariant of degree two, and ($iv$) `petter_10` computes $\sum_{1\leq i\leq n} i + 1$ and requires a polynomial invariant of degree two. Each benchmark takes two random inputs and we consider the following input ranges $\{[1, 10], [1, 100], [1, 1000]\}$. In total, we have 12 variants of `petter`, each run for values of `cbits` $\in \{5, 6, 7\}$—i.e., a total of 36 benchmarks.

*Program Repair* The third set of benchmarks contains 54 variants of SKETCH problems from the domain of automatic feedback generation for introductory programming assignments [7]. Each benchmark corresponds to an incorrect program submitted by a student and the goal of the synthesizer is to find a small variation of the program that behaves correctly on a set of test cases. We select the 6/11 benchmarks from the tool Qlose [7] for which ($i$) our implementation can support all the features in the program, and ($ii$) our data flow analysis identifies at least one variable that can be tracked modularly. Of the remaining benchmarks, 3/11 do not contain variables that can be tracked modularly, and 2/11 call auxiliary functions that cannot be translated into SKETCH. For each

Table 1: Effectiveness of different solvers. SAT (resp. UNSAT) denotes the number of benchmarks for which solver could find a solution to the benchmarks (resp. prove no solution existed) while TO denotes the number of timeouts.

| | | Polynomials | | | Invariants | | | Program repair | | |
|---|---|---|---|---|---|---|---|---|---|---|
| **Solver** | **Solved** | **SAT** | **UNSAT** | **TO** | **SAT** | **UNSAT** | **TO** | **SAT** | **UNSAT** | **TO** |
| Unary | **69**/181 | 12 | 4 | 65 | 5 | 0 | 41 | 48 | 0 | 6 |
| Binary | **127**/181 | 70 | 6 | 5 | 17 | 0 | 29 | 34 | 0 | 20 |
| Unary-p | **169**/181 | 73 | 5 | 3 | 41 | 2 | 3 | 48 | 0 | 6 |
| Unary-p-inc | **172**/181 | 73 | 6 | 2 | 41 | 2 | 3 | 50 | 0 | 4 |

program, we consider the original problem and two variants where the integer inputs are multiplied by 10 and 100, respectively. Further, for each program variants, we impose an assertion specifying that the distance between the original program and the repaired program is within a certain bound. We select three different bounds for each program: the minimum cost $c$, $c + 100$, and $c + 200$.

#### 8.2 Performance of Unary-p

Table 1 summarizes our comparison. First, we compare the performance of Unary-p and Unary. We use $\mathbb{P} = \{2, 3, 5, 7, 11, 13, 17\}$, which is enough for Unary-p to always find correct solutions (we verify the correctness of a solution by instantiating the hole values in the original sketch programs). Unary can only solve 69/181 benchmarks while Unary-p can solve 169/181. Figure 7a shows a scatter plot (log scale) of the solving times for the two techniques: each point below the diagonal line denotes a benchmark on which Unary-p was faster than Unary. Points on the extreme right-hand side of the plot denote timeout for Unary. When both solvers terminate, Unary-p (avg. 1.7s) is 6.1X (geometric mean) faster than Unary (avg. 25.0s).

Next, we compare the performance of Unary-p and Binary (Figure 7b). On the 64 easier benchmarks that Binary can solve in less than 1 second, Binary (avg. 0.55s) outperforms Unary-p (avg. 2.32s), but Unary-p still has reasonable performance. On the 49 benchmarks that Binary can solve between 1 and 10 seconds, Unary-p (avg. 3.5s) is on average 1.9X faster than Binary (avg. 6.9s). Most interestingly, for the 14 harder benchmarks for which Binary takes more than 10 seconds, Unary-p (avg. 5.7s) is on average 15.9X faster than Binary (avg. 90.9s). Remarkably, Unary-p *solved 43 of the benchmarks (in less than 8s each) for which* Binary *timed out*[4], and Unary-p only timed out for two benchmarks that Binary could solve in less than a second and one benchmark that Binary could solve in 260s. Finally, we would like to highlight that for 41/208 benchmarks, even Unary outperforms Binary. As expected from

[4] During our experiment, we observed that Binary *incorrectly* reported UNSAT for 10 satisfiable benchmarks. We reported these benchmarks as timeouts and have contacted the authors of Sketch to address the issue.

the discussion throughout the paper, these are benchmarks typically involving complex operations but not involving overly large numbers.

We can now answer **Q1**. First, **Unary-p consistently outperforms Unary** across all benchmarks. Second, **Unary-p outperforms Binary on hard-to-solve problems and can solve problems that Binary cannot solve**—e.g., Unary-p solved 28/46 invariant problems that Sketch could not solve. Unary-p and Binary have similar performance on easy problems.

*Comparison to full SMT encoding* For completeness, we also compare our approach to a tool that uses SMT solvers to model the entire synthesis problem. We choose the state-of-the-art SMT-based synthesizer Rosette [23] for our comparison. Rosette is a programming language that encodes verification and synthesis constraints written in a domain-specific language into SMT formulas that can be solved using SMT solvers.

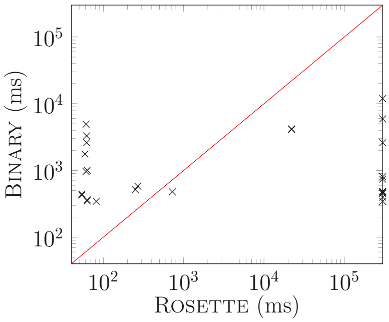

Fig. 6: Rosette vs Binary

We only run Rosette on the set of Polynomials because Rosette does support the theories of integers, but does not have native support for loops, so there is no direct way to encode Invariants and Program Repair benchmarks. To our knowledge, Rosette provides a way to specify the number $k$ it uses to model integers and reals as $k$-bit words, but the user has no control over how many bits it uses for unknown holes specifically. So we evaluate 27 instead of 81 variants of the polynomial synthesis problem on Rosette, i.e., we consider different numbers of cbits.

Figure 6 shows the running times (log scale) for Rosette and Binary with `cbits`=6. Rosette successfully solved 16/27 benchmarks and it terminates quickly (avg. 2.9s) when it can find a solution. However, Rosette times out on 11 benchmarks for which Binary terminates. The timeouts are due to the fact that Rosette employs full SMT encodings that combine multiple theories while Binary uses a SAT solver that is only modified to accommodate SMT-like integer constraints. Since we now know full SMT encodings are not as general and efficient as the encodings used in Sketch, we will only evaluate the effectiveness of our technique based on comparison with Binary.

Finally, we tried applying our prime-based technique to Rosette and, as expected, the technique is not beneficial due to the binary encoding of numbers in SMT, and causes all benchmarks to timeout. To summarize, ($i$) SMT solvers cannot efficiently handle the synthesis problems considered in this paper, and ($ii$) our technique is better suited for SAT solvers than SMT solvers.

#### 8.3 Performance of Incremental Solving

Our implementation of the incremental solver Unary-p-inc first attempts to find a solution with the prime set $\mathbb{P} = \{2, 3, 5, 7\}$. If the solver returns a correct

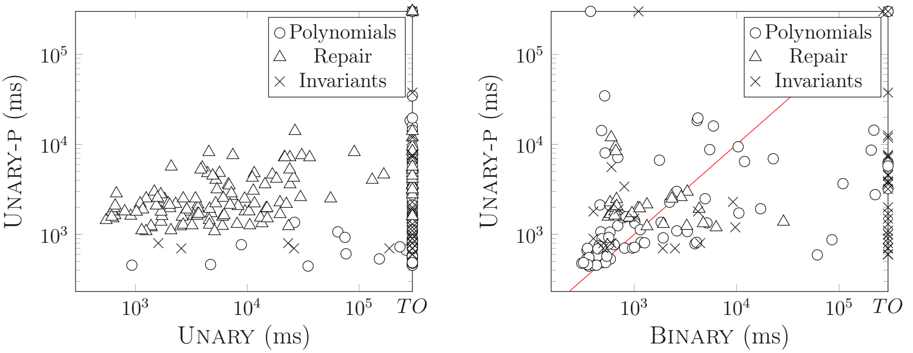

(a) UNARY vs UNARY-P

(b) BINARY vs UNARY-P

Fig. 7: Performance of UNARY, BINARY, and UNARY-P.

solution, UNARY-P-INC terminates. Otherwise, UNARY-P-INC incrementally adds the next prime to $\mathbb{P}$ until it finds a correct solution, it proves there is no solution, or it times out. UNARY-P-INC is 25.2% (geometric mean) slower than UNARY-P (Figure 8 (log scale)). UNARY-P-INC can solve three benchmarks for which both UNARY-P and BINARY timed out. To answer **Q3**, **UNARY-P-INC and UNARY-P have similar performance.**

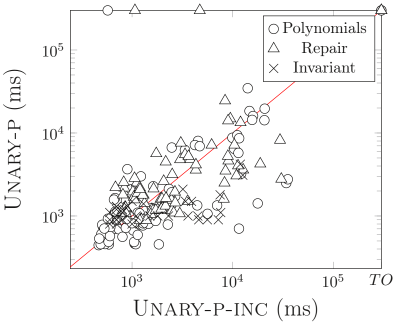

Fig. 8: UNARY-P-INC vs UNARY-P

#### 8.4 Varying the Prime Number Set $\mathbb{P}$

In this experiment, we evaluate how different prime number sets affect UNARY-P.

We consider the 5 increasing sets of primes: $\mathbb{P}_5 = \{2, 3, 5\}$, $\mathbb{P}_7 = \{2, 3, 5, 7\}$, $\mathbb{P}_{11} = \{2, 3, 5, 7, 11\}$, $\mathbb{P}_{13} = \{2, 3, 5, 7, 11, 13\}$, and $\mathbb{P}_{17} = \{2, 3, 5, 7, 11, 13, 17\}$. Figure 9a (log scale) shows the running times for all the polynomial benchmarks with `cbits`=7 (showing all benchmarks would clutter the plot). The points where the lines change from dashed to solid denote the number of primes for which the algorithm starts yielding correct solutions. As expected, a smaller set of primes leads to faster solving times as the resulting constraints are smaller and fewer bits are needed for encoding intermediate values. The runtime on average grows with the increasing size of the primes. For example, across all benchmarks, using $\mathbb{P}_{17}$ takes 23% longer on average than using $\mathbb{P}_{11}$. To answer **Q3**, UNARY-P is **slower when using increasingly large sets of prime**.

In terms of correctness, we find that smaller prime sets often yield incorrect solutions ($\mathbb{P}_5$ (37% correct), $\mathbb{P}_7$ (70%), $\mathbb{P}_{11}$ (86%), $\mathbb{P}_{13}$ (97 %), and $\mathbb{P}_{17}$ (100%) because there is not enough discriminative power with fewer primes and the

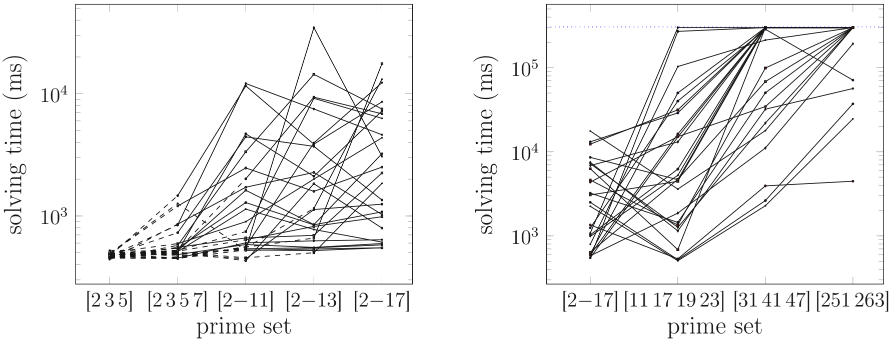

(a) Larger sets of primes

(b) Larger primes

Fig. 9: Performance for different sets of prime numbers.

solutions may overfit to the smaller set of intermediate values. It is interesting to note that even prime sets of intermediate size often lead to correct solutions in practice, which explains some of the speedups observed in the incremental synthesis algorithm. To answer **Q4**, **Unary-p is able to synthesize correct solutions even with intermediate sized sets of primes**.

*Changing Magnitude of Primes* We also evaluate the performance of Unary-p when using primes of different magnitudes. We consider the sets of primes $\{11, 17, 19, 23\}$, $\{31, 41, 47\}$, and $\{251, 263\}$, which define similar integer ranges, but pose different trade-offs between the number of used primes and their sizes—e.g., the set $\{251, 263\}$ only uses two very large primes. Since the different sets cover similar integer ranges, they all produce correct solutions. Figure 9b (log scale) shows the running time of Unary-p for the same benchmarks as Figure 9a. Larger prime sets of smaller prime values require less time to solve than smaller prime sets of larger prime values. This result is expected since, in the unary encoding of numbers, representing larger numbers requires more bits.

#### 8.5 Size of SAT Formulas

In this experiment, we compare the sizes of the intermediate SAT formulas generated by Unary-p and Unary. Figure 10a shows a scatter plot (log scale) of the number of clauses of the largest intermediate SAT query generated by the CEGIS algorithm for the two techniques. We only plot the instances in which Unary was able to produce at least a SAT formula. Unary produces SAT formulas that are on average 19.3X larger than those produced by Unary-p. To answer **Q5**, as predicted by our theory, **Unary-p produces significantly smaller SAT queries than Unary**.

*Performance vs Size of SAT Queries* We also evaluate the correlation between synthesis time and size of SAT queries. Figure 10b plots the synthesis times of both solvers against the sizes of the SAT queries. It is clear that the synthesis

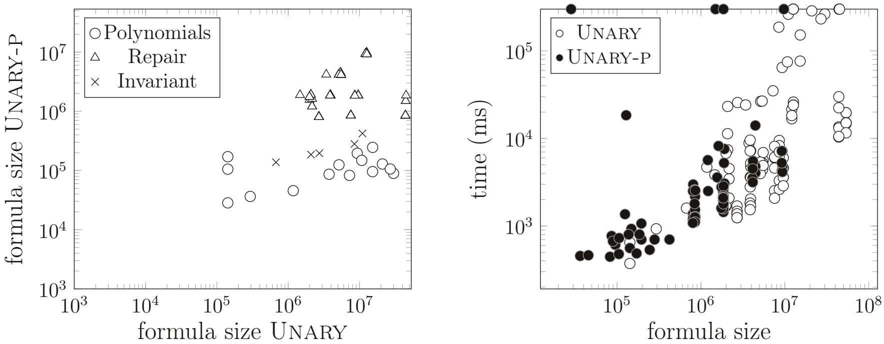

(a) Size: UNARY-P vs UNARY

(b) Performance vs size

Fig. 10: SAT formulas sizes and performance.

time increases with larger SAT queries. The plot illustrates how the solving time strongly depends on the size of the generated formulas.

### 9 Related Work

*Program Sketching* Program sketching was designed to automatically synthesize efficient bit-vector manipulations from inefficient iterative implementations [21]. The SKETCH tool has since been engineered to support complex language features and operations [19]. Thanks to its simplicity, sketching has found wide adoption in applications such as optimizing database queries [3], automated feedback generation [18], program repair [7], and many others. Our work further extends the capabilities of SKETCH in a new direction by leveraging number theory results. In particular, our technique allows SKETCH to handle sketches manipulating large integer numbers. To the best of our knowledge, our technique is the first one that can solve many of the benchmarks presented in this paper.

*Uses of Chinese Remainder Theorem* The Chinese Remainder Theorem and its derivative corollaries have found wide application in several branches of Computer Science and, in particular, in Cryptography [11, 26].

The idea of using modular arithmetic to abstract integer values has been used in program analysis. Since modular fields are finite, they can be used as an abstract domain for verifying programs manipulating integers [5]—e.g., the abstract domain can track whether a number is even or odd. Our work extends this idea to the domain of program synthesis and requires us to solve several challenges. First, when used for verifying programs, the modular abstraction is used to overapproximate the set of possible values of the program and does not need to be precise. In particular, Clark et al. [5] allow program operations that are in the `IMP` language but not in the `IMP-MOD` language and lose precision when modeling such operations—e.g., when performing the assignment $x = x/2$ the value of $x$ mod 2 can be either 0 or 1. Such imprecision is fine in program analysis since the abstraction is used to show that a program does not contain a bug—i.e., even in the abstract domain, the problem behaves fine. In our setting, the problem is opposite as we use the abstraction to simplify the synthesis problem and provide a theory for when the modular and integer semantics are equivalent.

*Pruning Spaces in Program Synthesis* Many techniques have been proposed to prune large search space of possible programs [14]. Enumerative synthesis techniques [24, 12, 13, 17] enumerate programs in a search space and avoid enumerating syntactically and semantically equivalent terms. Some synthesizers such as Synquid [16] and Morpheus [10] use refinement types and first-order formulas over specifications of DSL constructs to refute inconsistent programs. Recently, Wang et al. [25] proposed a technique based on abstraction refinement for iteratively refining abstractions to construct synthesis problems of increasing complexity for incremental search over a large space of programs.

Instead of pruning programs in the syntactic space, our technique uses modular arithmetic to prune the semantic space—i.e., the complexity of verifying the correctness of the synthesized solution—while maintaining the syntactic space of programs. Our approach is related to that of Tiwari et al. [22], who present a technique for component-based synthesis using dual semantics—where syntactic symbols in a language are provided two different semantics to capture different requirements. Our technique is similar in the sense that we also provide an additional semantics based on modular arithmetic. However, we formalize our analysis based on number theory results and develop it in the context of general-purpose Sketch programs that manipulate integer values, unlike Tiwari et al.'s work that is developed for straight-line programs composed of components.

*Synthesis for Large Integer Values* Abate et al. propose a modification of the Cegis algorithm for solving *syntax-guided synthesis* (SyGuS) problems with large constants [1]. SyGuS differs from program sketching in how the synthesis problem is posed and in the type of programs that can be modeled. In particular, in SyGuS one can only describe programs representing *SMT formulas* and the logical specification for the problem can only relate the input and output of the program—i.e., there cannot be intermediate assertions within the program. The problem setup and the solving algorithms proposed in this paper are orthogonal to those of Abate et al. First, we focus on program sketching, which is orthogonal to SyGuS as sketching allows for richer and more generic program spaces as well as richer specifications. While it is true that certain synthesis problems can be expressed both as sketches and as SyGuS problems, this is not the case for our benchmarks programs, which use loops, arrays and non-linear integer arithmetic, all of which are not supported by SyGuS. Second, our technique is motivated by how Sketch encodes and solves program sketches through SAT solving. While the traditional Sketch encoding can explode for large constants, the same encoding allows Sketch to solve program sketches involving complex arithmetic and complex programming constructs. The algorithm proposed by Abate et al. iteratively builds SMT (not SAT) formulas that are required to be in a decidable logical theory. Such an encoding only works for the restricted programming models used in SyGuS problems.
